# Skilled performance tests and their use in diagnosing handedness and footedness at children of lower school age 8–10

**DOI:** 10.3389/fpsyg.2014.01513

**Published:** 2015-01-12

**Authors:** Martin Musalek

**Affiliations:** Faculty of Physical Education and Sport, Charles UniversityPrague, Czech Republic

**Keywords:** handedness, footedness, performance tests, laterality, children, fine motor, gross motor

## Abstract

Previous research has shown that hand and foot preferences do not develop in parallel in children and it has been discovered that in children foot preference stabilizes later. Therefore, the aim of this study is to verify whether the differences in stabilization will also be manifested through less consistent results of selected skilled foot performance tests in a comparison with selected skilled hand performance tests. A total of 210 8–10 year old children from elementary schools were recruited for this study. Hand and foot preferences were first tested using hand and foot preference observable measure tasks; consequently, all participants performed four skilled hand performance tests and three foot performance tests. Unlike in complex skilled hand performance tests, which showed a significant convergent validity 0.56–0.89 with hand preference tasks, in complex skilled foot performance tests a very low convergent validity 0.25–0.46 with foot preference tasks was detected. The only skilled foot performance indicator which showed an acceptable convergent validity with foot preference tasks was the “foot tapping” test 0.65–0.85, which represents rather a gross motor activity. Moreover, further results of the tests suggest that complex or fine motor performance tests used for diagnosing laterality of the lower limb that have a manipulative character probably do not represent suitable indicators for children in the given age category. The same trend was revealed in both females and males. This indicates that the level of laterality assessed as difference in skilfulness between the preferred and the non-preferred limb in children in the given age group probably develops in the same way in both genders.

## INTRODUCTION

Numerous studies have in the past been dedicated to human laterality, which represent a multidimensional trait (Corballis, 2010). It is well known, for instance, that in the adult population 90% of people prefer to use their right hand for common manual tasks, whereas about 10% of the population are so called lefthanders (Annett, 1994; Raymond et al., 1996; Bryden et al., 1997; McManus, 2002). Another important finding is that throughout human life, the development of laterality is a very active process affected by both genetic and environmental factors (see: Porac et al., 1980; Geschwind and Galaburda, 1987; Halpern and Coren, 1991; Annett, 2002; McManus, 2002).

Research into development of laterality in children has shown that it has different phases with respect to ontogenesis. The authors McManus et al. (1988) suggested that handedness in children is generally defined by one basic factor and begins to become fixed around the age of 3; it becomes stabilized and its level increases between 3 and 7 years of age. According to the authors, stabilization gradually weakens between 7 and 9 years of age (McManus et al., 1988). Studies of Cavill and Bryden (2003) or Whittington and Richards (1987) have proven that the development of handedness (right or left) can be determined in children relatively early; which is not entirely true for strength and consistency of handedness (De Gostini et al., 1992). Development of consistency and the level of preference of upper limbs in children has also been studied by authors using so called reaching tasks (e.g., Bryden and Roy, 2006; Carlier et al., 2006), which focused on whether a child would also manipulate with a tool using the preferred upper limb in the case that the tool was placed counter-laterally to the preferred hand. The conclusions of these studies showed that in this kind of motor activity 6- to 10-year-olds children demonstrate significantly more stable consistency of upper limb preference than younger children (Bryden and Roy, 2006; Carlier et al., 2006). Leconte and Fagard (2004), who also used the reaching task approach, revealed that consistency of handedness in children changes with the complexity of the activity which the child is forced to do with his/her upper limb. The authors also add that development of strength and consistency of handedness in children represents an important dynamic process (Leconte and Fagard, 2004).

In comparison with the number of studies on development of the upper-limb laterality, less research has been done into the development of footedness. Coren et al. (1981) found that 3- to 5-year-olds children, as well as a selected population of high-school students, demonstrated a significant right-hand preference. Their findings at the same time revealed that pre-school children had significantly less distinct lower limb preference (Coren et al., 1981). Studies by Gabbard et al. (1991); Gabbard (1992), and Gentry and Gabbard (1995) also found that foot preference in 3- to 5-year-olds children is much less consistent than hand preference. On average, the agreement of upper and lower limb preference in right-handers was 67% while in left-handers it was only 17%. According to the authors, significant stabilization of lower limb preference happens later, between 8 and 11 years of age (Gabbard, 1992; Gentry and Gabbard, 1995). A review study by Gabbard and Iteya (1996) also revealed that in 3- to 5-year-olds mix-footedness appears with twice as much occurrence as mix-handedness (Gabbard and Iteya, 1996). By contrast, Gudmundsson (1993), who studied conformity between upper and lower limb preference in pre-school and younger school children aged 3–11, found 85 and 87% conformity, respectively (Gudmundsson, 1993).

In the diagnosis of laterality, according to Corballis (2009), for example much less attention is paid to the detailed skilled performance approach (Corballis, 2009). The above-mentioned diagnosis of preference allows only a very limited detailed expression of strength of handedness or footedness. Consequently, in past decades performance tests have been created and verified which primarily focus on the difference between the upper limbs in performing the same motor tests (Scharoun and Bryden, 2014). Research has shown that in both children and adults the different skilfulness in terms of speed, precision or correctness of execution of motor activities strongly corresponds with upper limb preference (Peters, 1976; Annett et al., 1979; Rigal, 1992; Carlier et al., 1993; Cornish and McManus, 1996; Nalcaci et al., 2001). Nevertheless, it has also been found that the level of correspondence between preference and performance depends, to a great extent, on the type of performance test. In this context, Annett (1992) observed that the more the activity is of a fine motor character, the more significant the higher skilfulness of the preferred upper limb (Annett, 1992). The authors Roy et al. (1994) and Sainburg and Kalakanis (2000) later added that this considerably higher skilfulness of the preferred upper limb is observed primarily in motor activities in which higher demands are put on: (1) coordination and (2) integration of more segments of the limb, involved in the activity (e.g., shoulder and elbow joints; Roy et al., 1994; Sainburg and Kalakanis, 2000).

Performance tests to evaluate different skilfulness have also been created for lower limbs (see Knights and Moule, 1967; Beling et al., 1998). However, they showed congruency with the determined foot preference solely in the adult population.

In connection with laterality assessment, Rigal (1992), Steenhuis (1999) and Corey et al. (2001) have suggested that for reliable diagnostics, both preference indicators and performance tests should be used because laterality in humans does not represent a unidimensional trait (Rigal, 1992; Steenhuis, 1999; Corey et al., 2001). Even though previous studies focused on the development of upper and lower limb laterality, they mostly assessed development of handedness and footedness and their stability in the child population. The conclusions of studies focused on the question of consistency of upper and lower limb preference in child population suggest that stabilization of lower limb preference represents in children a longer process than stabilization of hand preference (see Gabbard, 1992; Gentry and Gabbard, 1995; Gabbard and Iteya, 1996). However, in connection with this finding we have observed that there are not enough studies attempting to verify whether later stabilization of the lower limb preference is also manifested in the results of performance tests for lower limbs that are used to diagnose laterality in the child population. The results of our previous research have suggested that primarily complex skilled foot performance tests do not show the differences between the preferred and the non-preferred lower limb with sufficient accuracy in 8- to 10-year-olds. In the monograph Musálek (2013), three identical performance tests (for the lower limb) were modeled using the confirmatory factor analysis for the population of 8- to 10-year-olds and for 17- to 19-year-old adolescents. These were complex motor activities which integrated more systems: (1) moving a small object by the lower limb in a limited space and (2) slalom with a tennis ball between obstacles and (3) an activity which focused primarily on speed while performing a simple task – foot tapping. The revealed results were extremely interesting. While in the adolescent population (17- to 19-year-olds), both complex tests had acceptable factor loadings in range: 0.61–0.72 for the modeled factor “foot performance,” in 8- to 10-year-olds factor loadings of the tests significantly lower in a range between 0.38–0.43 with respect to the “foot performance” factor. On the other hand, the foot tapping test showed a strong relationship to the “foot tapping” factor in both children and adolescents with factor loads 0.84 and 0.92, respectively. It was also revealed that loads of both complex skilled foot performance tests used in this study were for both children and adolescent populations significantly lower in comparison with complex fine motor tests for the upper limb (“spiral tracing,” “dot-filling”; Musálek, 2013). This result could be found due to the fact that upper limbs are primarily designed for manipulation, whereas lower limbs have primarily a postural function (Woodburne and Burkel, 1994, p. 87; Christou et al., 2003; Palastanga and Soames, 2011, p. 202). Therefore, based on this information we assume that in the given category of 8- to 10-year-olds skilled foot performance tests (spiral tracing by small cube; while standing, slalom with ball between obstacles) will show fewer differences and more inconsistencies (i.e., weaker lateralization) than complex tests designed for the upper limbs.

Moreover, a number of studies have also revealed that significant differences exist between males and females concerning consistency of handedness – it has been revealed that there is a significantly higher number of mixed-handers among males (e.g., Whittington and Richards, 1987; Sommer et al., 2008; Johnston et al., 2009). From the point of view of ontogenesis, a very interesting difference between males and females has been revealed in the strength of neural pathways leading into the cerebellum. These pathways which are involved, among other things, in realization of fine motor skills are according to Gurian et al. (2001) significantly stronger in females. Therefore, the second question studied in this research is whether the level of laterality assessed as difference in skilfulness between the preferred and the non-preferred limb will differ in males and in females. We suppose that such difference might be revealed in the form of a different level of relationship factor loadings – in selected performance tests to modeled factors: (1) hand performance, (2) foot performance.

## MATERIALS AND METHODS

### PARTICIPANTS

A total of 210 typically developing 8- to 10-year-olds (*n* = 107 males and *n* = 103 females; *M*_age_ = 9.1, SD = ± 0.78) from the Czech Republic were recruited for the current study. All participants were pupils of state primary schools in the capital Prague. The selection of the research file was done using the intentional selection process method. The following criteria for selection of participants were used:

(1) pupils were chosen only from schools which had a similar number of pupils in the given age category,
(2) only schools without any specific specialization (e.g., technical, artistic, sport, or linguistic) were selected,
(3) schools and classes with integrated children with special needs were not included in the selection.

As this study draws on the research (validation of variables for diagnosing of motor manifestations of laterality) performed at these selected schools in 2011 and published in 2013 (Musálek, 2013), all participants were chosen from the same schools, as in the previous research. We decided on this concept of an intentional selection process method in order to ensure maximum homogeneity of the file with respect to the findings of 2011.

The 8–10 age category was selected because at this age children’s motor skills are harmoniously developed with stable coordination patterns and this age is called the golden age of skill motor development (Ljach, 2002).

Ethics approval was granted by the Ethics Commission of the Faculty of Physical Education and Sport, Charles University. In addition, parental consent was obtained for all individuals.

### APPARATUS

In order to verify whether the selected skill performance tests really detect a difference in performance of the preferred and the non-preferred upper and lower limbs, the results of the seven selected skilled performance tests were first correlated with the results of seven observable preference measure tasks (four for handedness, three for footedness). The indicators used for evaluation of hand preference and foot preference have been validated for the Czech child population aged 8–10. Factor loads of hand preference indicators in a range: λ = 0.85–0.93, generic reliability McDonald ω = 0.95; factor loads of foot preference indicators in a range: λ = 0.66–0.90, generic reliability McDonald ω = 0.81 (Musálek, 2013). The results of the preference observable measure tasks also served to determine the preferred and the non-preferred limb as a necessary precondition for the selected skilled performance tests to be carried out in accordance with the given rules. Six of the seven observable preference measure tasks have already been used in previous research where theses indicators were approved as valid and reliable either as questionnaire items or preference tasks (e.g., Annett, 1970; Barnsley and Rabovitch, 1970; Oldfield, 1971; Sharman and Kulhavy, 1976; Tapley and Bryden, 1985; Rigal, 1992; Coren, 1993; Bishop et al., 1996; Doyen and Carlier, 2002; Mamolo et al., 2006).

At the same time, all seven performance tests were validated for the Czech child population aged 8–10 years. Five of them – four for handedness: (1) spiral tracing, (2) dot-filling, (3) tweezers and beads, (4) twisting box; and one for footedness: foot tapping – had an acceptable level of factor validity with respect to the modeled factors: (1) hand performance λ = 0.58–0.82. and (2) foot performance λ = 0.92. Subsequently approximated generic reliability of the tests modeled only under one factor “Performance of locomotive organs” had value McDonald ω = 0.83. These five tests have already been replicated in the study Scharoun et al. (2013) for the assessment of different performance of the preferred and the non-preferred upper and lower limbs in children with ADHD and their neurotypical controls. The results of this study revealed that all five performance tests are sufficiently sensitive to determine the performance of the preferred and the non-preferred limb and to detect motor problems in children with ADHD (Scharoun et al., 2013).

Preference strength was determined based on laterality quotient calculation, for which equations from previous studies were used (e.g., Humphrey, 1951; Harris, 1958; Bryden et al., 2007; Kalaycioğlu et al., 2008). Each execution in preferential tasks was marked 1 when right limb was used and 0 when the left limb was used.

Laterality quotient for the upper and lower limbs was calculated using the formula

$$LQ=\frac{R-L}{R+L}*100$$

#### Hand preference

***Throwing on target*.** The aim of the participant who sits on chair was to throw the foam ball with 58 mm in diameter using one hand to the target which was placed 2 m from participant. Task was repeated three times.

***Ring by bell*.** The examiner places a (metal) bell on the desk in front of the participant so that there was the same distance to both his/her hands. The aim of the participant was to take the bell in one hand and ring it.

***Card reaching task (Bishop task)*.** This task included A3 sheet of paper, divided in half by a vertical line The paper contains seven rectangular boxes with the dimensions of 6 cm × 3 cm forming a semicircle. There were seven cards in total in the boxes on the paper, each card having a different, clearly distinguishable color. Each box had its own description: the first box on the left was marked –3 on the shorter side, the second on the left was marked –2, etc., and the last box on the right was marked +3. The middle box on the axis of the paper was marked 0. The aim of the participant was to turn the card with the required color using one hand. The examiner first chooses the color of the card that s/he placed in the box marked 0. If the participant turned this card using the right hand, the examiner required him/her to turn the colored cards in the boxes marked in the following order: +2, –2, +3, –3. If the participant turned this card using the left hand, the examiner required him/her to turn the colored cards in the boxes marked in the following order: –2, +2, –3, +3. Examinator recorded frequency of using right hand or left hand, respectively.

***Erasing*.** The examiner places an erasing rubber with the dimensions of 4.5 cm × 2.5 cm on the desk in front of the participant so that both hands of the participant were in the same distance. Then the examiner asked the participant to erase the prepared drawn line.

#### Foot preference

***Kick to the ball on target*.** The aim of the participant was to kick the foam ball with 58 mm in diameter in order to hit the wooden block with an edge length of 40 mm placed 2 m from the ball. The kick was performed three times. After each attempt the examiner returned the ball to its original position.

***Using one foot, tap the rhythm that I am clapping*.** The participant sit on a chair in free space. The examiner claps a simple rhythm with a maximum of five claps. The task of the participant was to tap this rhythm on the floor using one lower limb.

***Perform jumps forward using one leg*.** The task of the participant was to perform jumps forward on one leg from examiner to definite point. It was done twice by participant.

#### Skilled hand performance tests

***Spiral tracing*.** The score sheet contains pre-drawn white spirals of the same shape and length in two gray square boxes with a side length of 50 mm. The largest diameter of the spirals was 41 mm, the thickness (width) of the spiral being 2 mm. The spiral in the right box is intended for the action of the right hand, and the spiral in the left box for the action of the left hand. The aim of the participant was to draw a spiral in the designated area of the spiral-shaped image, from the outer edge to the center. The position of the score sheet hadn’t to be changed during the entire test. An error, penalization 2 s, was noted when the participant left the designated area while drawing. This task was completed by non-preferred and preferred hand. The examiner recorded the final time after each drawing.

***Dot-filling*.** There were two boxes with circles on the inside page of the score sheet. The circles in left box are intended for the action of the left hand, and the circles in the right box are intended for the action of the right hand. Each of the boxes contains 90 identical circles. The diameter of a circle is 2 mm. The aim of the participant was to mark dots in the circles, in order to place the dot within the circle in the specified time of 30 s. Only those marks within the circles were counted toward performance. Task was completed by non-preferred and preferred hand.

***Moving beads from one box into another using tweezers*.** This task included two open matchboxes behind each other and a pair of tweezers with a length of 150 mm on the desk in front of the participant so that there is the same distance between both his/her hands and the closer matchbox; the closer matchbox contains 20 beads with 5 mm in diameter, and the second is empty. The aim of the participant was to move the beads one by one from the full box to the empty box using the tweezers in the specified time of 30 s. The task was completed with the preferred and non-preferred hand, where the number of beads transported in 30 s was recorded.

***Turning a box alternately with the front and the rear side on the table*.** This task included a closed empty matchbox with the front facing upward in front of the participant at the midline. The aim of the participant was to turn the matchbox using one hand by its front and back alternately faces the desk. The matchbox had to always touch the desk with one of its parts, i.e., the matchbox hadn’t to be lifted from the desk. The task was completed with the preferred and non-preferred hand, where the number of turns in 30 s was recorded.

#### Skilled foot performance tests

***Foot tapping*.** For this task, the participant stood next to a desk, with the preferred leg closest to the desk The aim of the participant was to perform tapping in a standing position for 30 s using a lower limb so that the motion is performed in the sagittal plane. The participant taught the ground in front of him/her with the heel and the ground behind him/her with the tip, the range of the motion being the length of one foot of the participant. The task was completed with the preferred and non-preferred foot, where the number of tapps in 30 s was recorded (Musálek, 2013).

Unlike the “foot tapping” test, the following two tests (slalom with a ball between obstacles and spiral tracing by small cube) proved valid for adolescent population of selected students from the Czech Republic; however, this is not true for children aged 8–10 (Musálek, 2013). Also due to the previous equivocal results, we used the following skilled foot performance tests in this study: (1) slalom with a ball between obstacles and (2) spiral tracing by small cube; this test was derived from two tests – spiral tracing test used for hand and moving a cube in the “maze” while standing performed by foot. Both tests underwent multiple content validation with instructions and technical parts (tools) adapted so that different performance of the preferred and the non-preferred lower limb could be assessed.

The content validity of both tests was assessed by six selected experts in: anthropology, kinesiology, psychology, motor development, special pedagogy, and neurology.

***While standing, slalom with ball between obstacles*.** This task included eight cubes on the floor in the line. Distance between each two cubes was 10 cm. In distance 15 cm in front of first cube and 15 cm behind last cube was on the floor attached color line. The aim of the participant was to performed slalom with tennis ball with 65 mm in diameter between cubes. Participant could move ball between obstacles only from top. Each contact of the ball and obstacle is error. This error was counted as 2 s penalty. Participant had to go through whole track from line to line. The task was completed with the preferred and non-preferred foot.

***Spiral tracing by small cube*.** This task included A3 sheet of paper which had a spiral drawn on both sides on the floor. The spiral on each side of the paper was 30 cm in diameter with the thickness (width) of the spiral being 4 cm. The aim of the participant was to use the cube provided with a width of 1.5 cm to copy the spiral path in the designated area of the spiral-shaped image, from the outer edge to the center. The spiral had to be copied only by moving the cube by imposing pressure on the side of the cube; it was therefore forbidden to manipulate the cube by placing the sole of the foot on the top of it. An error, penalization 2 s, was noted when the participant left the designated area while copying. This task is completed by non-preferred and preferred foot. This motor test was carried out without preparation. The examiner recorded the final time after each copying.

### STATISTICAL ANALYSIS

In order to determine the level of the relationship between the selected preference measure observable tasks and skilled performance tests, biserial and polyserial correlations were used. Consequently, difference in performance of the preferred and the non-preferred limb for each skilled performance indicator were assessed by a paired *t*-test with the level of statistical significance *p* < 0.05 and substantive significance Cohen *d* > 0.7 (Cohen, 1988). In order to determine possible differences in the structure of hand and foot performance in females and males, the confirmatory factor analysis multigroup modeling approach was used (Muthén and Muthén, 2010). Robust maximum likelihood (Ferron and Hess, 2007; Muthén and Muthén, 2010) was used as the estimate parameter because in our case the multivariate normality of data condition was not fulfilled. The data analysis was done using the statistical software M-Plus 6 (Muthén and Muthén, 2010) and NCSS2007 (Hintze, 2007).

## RESULTS

First we analyzed the number and ratio of those that were pronounced: right-sided children, left-sided children and children who at least once changed limbs while performing the preference tasks (see Methods apparatus). Of the 210 participants, 136 children had uncrossed lateral preferences (right-handed and right-footed), (65 males and 71 females) LQ = 100, and 18 children had uncrossed lateral preferences (left-handed and left-footed), (10 males and 8 females) LQ = 0. The LQ of the remaining 56 children (32 males and 24 females) ranged within LQ = 31.25–75.

The subsequent correlation analysis between preference observable measure and skilled hand performance tests is shown in **Table 1**. The tests which detect different skilfulness of the preferred and the non-preferred upper limb manifest sufficient convergent validity with hand preference observable measure: correlation in a range *r* = 0.56–0.89. It follows that the selected hand performance tests have a sufficient capacity to adequately determine the difference between the preferred and the non-preferred upper limb.

**Table 1:** Convergent validity between hand preference observable measure and hand performance tests.

Item	Throwing	Ring the bell	Bishop task	Erasing
Spiral tracing	-0.89	-0.84	-0.66	-0.82
Dot-filling	0.70	0.66	0.64	0.63
Twistbox	0.78	0.75	0.64	0.66
Tweezers and beads	0.75	0.70	0.58	0.56

On the other hand, **Table 2**, shows that the correlation analysis between foot preference observable measure and skilled foot performance tests revealed that two of three performance tests (slalom between obstacles and spiral tracing with small cube) do not manifest a satisfactory convergent validity *r* = 0.25–0.46 with foot preference observable measure. This finding suggests that fine motor or complex tests for lower limbs lack sufficient sensitivity to distinguish between the preferred and the non-preferred lower limb in the given age group.

**Table 2:** Convergent validity between foot preference tasks and foot performance tests.

Item	Kicking	Tapp rhytmus	Hop forward
Slalom with ball between obstacles	-**0.46**	-**0.33**	-**0.26**
Spiral tracing by small cube	-**0.39**	-**0.43**	-**0.25**
Foot tapping	0.85	0.74	0.65

*Correlations lower than 0.50 are shown in boldface*.

Next we assessed the capacity of the seven skilled performance tests to determine preferred and non-preferred upper and lower limb by significance of difference in skilfulness of the preferred and the non-preferred limb.

**Table 3** shows that all the skilled hand performance tests used were able to significantly determine the difference between skilfulness of the preferred and the non-preferred upper limb, with the preferred upper limb being significantly more precise and quicker *p* < 0.05, *Cohen d* in the tests *d* = 0.84–2.91. On the contrary, the same cannot be said about the results of the foot performance tests. Among them, only the “tapping” test showed significant capacity to determine the difference in skilfulness of the preferred and the non-preferred lower limb *p* < 0.05 and *Cohen d*, *d* = 1.22. The other two tests, which were of a complex motor character, with the “slalom with a ball between obstacles” test having extra demands on balance, did not confirm the significance of the different performance of the preferred and the non-preferred lower limb *Cohen d* ranging within *d* = 0.22–0.27. These results together with findings regarding convergent validity for the lower limb (see **Table 2**) support the hypothesis that fine motor or complex tests for diagnosing lower limb laterality in children of the given age category are not suitable due to their low discrimination capacity between the preferred and the non-preferred lower limb.

**Table 3:** Differences in performances between preferred and non-preferred hand in skilled hand performance tests.

Item	M NP – limb	SD NP – limb	M P – limb	SD P – limb
**Hand performance tests**
Spiral tracing	79.3 s	23.4	44.2* s	13.8
Dot-filling	12.2 dots	5.1	34.3* dots	8.3
Tweezers and beads	7.9 beads	1.7	12.1* beads	2.1
Twistbox	38.4 twists	4.9	43.3* twists	5.1
**Foot performance tests**
Slalom with ball between obstacles	53.7 s	17.8	50.9 s	16.6
Spiral tracing by small cube	43.7 s	16.4	42.1 s	15.8
Tapping foot	32.4 tapps	7.1	41.2* tapps	7.3

NP – limb, non-preferred limb; P – limb, preferred limb; *significant difference between performance of non-preferred and preferred limb *p* < 0.05.

Next, we modeled all skilled performance tests in two-factor structure in order to determine whether the relationship between the individual indicators and defined latent variables upper limb performance and lower limb performance do not differ significantly in females and males.

The multigroup model assessed whether the child’s gender in the given age group does not represent a significant factor in the process of lateralization. A two-factor model for females and males shows that factor load does not differ significantly for most items. **Table 4** also shows that most indicators detected laterality (differences in skilfulness of the preferred and the non-preferred limb) between males and females aged 8–10 with approximately the same strength. The “foot tapping” performance test was the only exception, revealing significant difference between factor load in males, *r* = 0.56, and females, *r* = 0.74 at the significance level of *p* < 0.05. There could be two reasons for this result. Firstly, possibly in males stabilization of lower limb performance takes longer. Or secondly, that on the contrary the smaller difference in performance of the right and the left lower limb is caused by the relationship between the character of the test and a certain environmental factor.

**Table 4:** **Factor loadings of the 2-factor model – factors: (1) upper limb performance and (2) lower limb performance**.

Factors and used performance tests	Male		Female	
	λ	Uniq	λ	Uniq
**Upper limb performance factor**
Spiral tracing	-0.84	0.25	-0.78	0.43
Dot-filling	0.78	0.39	0.87	0.24
Twistbox	0.63	0.56	0.67	0.55
Tweezers and beads	0.47	0.76	0.58	0.67
**Lower limb performance factor**
Slalom with ball between obstacles	-0.32	0.86	-0.38	0.74
Spiral tracing by small cube	-0.30	0.89	-0.26	0.92
Tapping foot	0.56*	0.69	0.74*	0.44

Names of factors are in boldface; λ, factor loading; Uniq, uniqueness - residual variance; *significant difference between factor loadings *p* < 0.05.

## DISCUSSION

The aim of the study was to verify in a selected child population whether later stabilization of lower limb preference in comparison to hand preference determined in children (Coren et al., 1981; Gabbard et al., 1991; Gabbard, 1992; Gentry and Gabbard, 1995) is also manifested in lower consistency of performance test results for lower limbs used for the diagnosis of laterality. Within this question we have further studied whether the speed of lateralization diagnosed by selected indicators differs with respect to gender.

First, diagnosis of upper and lower limb preference was carried out using validated measure observable tasks.

Polyserial correlation between all selected skilled hand performance tests and hand measure observable task clearly demonstrated significant convergent validity ranging within *r* = 0.56–0.89. On the other hand, very weak correlations with foot preference ranging within *r* = 0.25–0.46 were determined in polyserial correlation between foot preference tasks and skilled foot performance tests in “slalom between obstacles” and “spiral tracing with small cube” tests. Consequently, convergence was not confirmed for two-foot performance tests and preference tasks, which suggests that lower limb lateralization in children is probably not identical in strength with upper limb lateralization. *t*-test results showed that selected indicators, which have also been validated for the Czech population, assessing upper limb preference in 8- to 10-year olds determine the difference between the preferred and the non-preferred upper limb *p* < 0.05 very well, with the non-preferred upper limb always being slower and less precise. This is in line with the conclusions of studies demonstrating that different skilfulness in speed, precision and correctness of execution of the motor activity strongly corresponds with the preferred upper limb in children (i.e., Annett et al., 1979; Rigal, 1992; Carlier et al., 1993; Cornish and McManus, 1996; Nalcaci et al., 2001). Moreover, these results also correspond with the conclusions of studies (Whittington and Richards, 1987; McManus et al., 1988; Cavill and Bryden, 2003; Bryden and Roy, 2006; Carlier et al., 2006) which show that between 6 and 10 years of age stability of hand preference in children is quite firm. In this respect it was also proved that the finer the motor activity, the bigger the differences between the performance of the preferred and the non-preferred upper limb, which confirms the arguments of Annett (1992). The biggest differences between performance of the preferred and the non-preferred upper limbs were found in complex tests with high demands on coordination (“spiral tracing” and “dot-filling”). This supports hypotheses made by Roy et al. (1994) and Sainburg and Kalakanis (2000) or Scharoun et al. (2013). They claim that significantly higher skilfulness of the preferred upper limb is observed in activities in which more segments of the given limb (e.g., shoulder and elbow joint) are involved at the same time (Roy et al., 1994; Sainburg and Kalakanis, 2000; Scharoun et al., 2013).

However, the results of the performance tests selected for the lower limb did not clearly detect a difference in skilfulness of the preferred and the non-preferred lower limb and thus confirmed problems detected with convergent validity in some skilled foot performance tests. Two out of three tests used (“slalom between obstacles” and “spiral tracing with small cube”), which compared to the tapping test by lower limb are more complex and have a finer motor character, revealed insignificant differences in performance between the preferred and the non-preferred lower limb. It is interesting to note that the “slalom between obstacles test” is validized in the CR for the adult population, in which no problems appeared in detecting difference in performance of the preferred and non-preferred lower limb. These findings are in conformity with studies (Knights and Moule, 1967; Beling et al., 1998) which revealed agreement of results of performance tests with determined foot preference solely in the child population. The revealed low sensitivity of complex and fine motor laterality performance tests for lower limb in children could be related to the detected longer stabilization process of the lower limb preference (Coren et al., 1981; Gabbard et al., 1991; Gabbard, 1992; Gentry and Gabbard, 1995; Gabbard and Iteya, 1996). This shows that lower limb performance in children is limitary. Paradoxically, too fine motor tests or too complex tests with high demands on coordination cannot determine the difference between the preferred and the non-preferred lower limb based on the results. Finally, we verified whether the lateralization process of the upper and lower limbs assessed by performance tests happens differently for females and males at this age. A two-factor model where all seven skilled performance tests were tested showed that the sensitivity of the selected indicators for detecting laterality of the upper and lower limbs is quite similar for both genders. This means that the lateralization process for the upper and lower limbs is probably quite similar in females and males at this age. The only difference of some significance revealed was related to factor load of the “foot tapping” test in females *r* = 0.74 and males *r* = 0.56 with factor validity coefficient for females being significantly *p* < 0.05 higher in comparison with factor validity of this indicator in males. This difference might be explained by some environmental factors, in males primarily by collective sports where both lower limbs are used (e.g., football). Consequently, the “foot tapping” test might not be sensitive enough to determine the difference between the preferred and the non-preferred lower limb in males. On the other hand, in females, who are not affected by these environmental factors, or are affected to a much smaller extent, the “foot tapping” test determined the difference between the preferred and the non-preferred lower limbs very well.

## CONCLUSION

It was revealed that in skilled hand performance tests, the more complex and more demanding in terms of coordination the motor activity is, the bigger the differences there are between the preferred and the non-preferred upper limb. However, the same result was not proved in skilled foot performance tests. On the contrary, the more demanding the lower limb tests were, the worse the convergence validity of these tests in connection to preference tasks. This finding in children could be related to a longer stabilization process of the lower limb preference (see Coren et al., 1981; Gabbard et al., 1991; Gabbard, 1992; Gentry and Gabbard, 1995). It is interesting to note that the lateralization process assessed by difference in performance in skilled performance tests happens in parallel in both genders.

## Conflict of Interest Statement

The author declares that the research was conducted in the absence of any commercial or financial relationships that could be construed as a potential conflict of interest.
